# VISUAL IMPAIRMENT PREDICTS PHYSICAL FUNCTION DECLINE: THE HEALTH, AGING, AND BODY COMPOSITION (HEALTH ABC) STUDY

**DOI:** 10.1093/geroni/igac059.537

**Published:** 2022-12-20

**Authors:** Atalie Thompson, Michael Miller, Christopher Webb, Jeff Williamson, Stephen Kritchevsky

**Affiliations:** Atrium - Wake Forest Baptist Health, Winston-Salem, North Carolina, United States; Wake Forest School of Medicine, Winston-Salem, North Carolina, United States; Atrium Health Wake Forest Baptist, Winston Salem, North Carolina, United States; Wake Forest School of Medicine, Winston-Salem, North Carolina, United States; Wake Forest School of Medicine, Winston-Salem, North Carolina, United States

## Abstract

The relationship between visual impairment (VI) and decline in physical function with age is poorly understood. We constructed separate linear mixed models to evaluate the relationship of self-reported (visual function question (VFQ) score) or performance-based (visual acuity (VA); log contrast sensitivity (LCS); stereoacuity (SA)) VI with change in performance on the Short Physical Performance Battery (SPPB) over 8 years in 2219 Health ABC participants. Mean age was 75.5 years (range 71-82); 52.4% were female, and 37.4% were black. For all measures of visual function, better vision was associated with loss of approximately -0.3 SPPB units/year which was similar to the unadjusted change in SPPB over time (-0.328 units/year 95%CI (-0.35, -0.31)). Participants with LCS ≤1.3 log units experienced 58% faster rate of decline versus those with better LCS (test of difference in slopes p< 0.0001). Those with poor VA ≥20/50 showed a 50% greater decline in SPPB (p=0.0029), and those with low SA ≤85 arcsec demonstrated a 33% faster decline (p< 0.001) relative to those with better visual function. Compared to the slope at the mean VFQ score, a 1 standard deviation lower score was associated with 23% greater decline in SPPB (p< 0.0001). The difference in SPPB slopes remained significant across VI measures after adjusting for longitudinal decline associated with age, sex, and black race (all p< 0.05). Both self-reported and performance-based VI predicted faster declines in SPPB over time. Whether older adults with VI might benefit from targeted intervention to prevent declining mobility function remains to be evaluated.

